# Gold Foil in Anterior Bridges

**Published:** 1920-08

**Authors:** F. H. Entriken

**Affiliations:** Walla Walla, Wash.


### GOLD FOIL IN ANTERIOR BRIDGES

BY F. H. ENTRIKEN, D.M.D., WALLA WALLA, WASH.

Research of the past few years in regard to devitalizing teeth and the resulting focal infection makes it imperative that the construction of bridges should be as conservative as possible of the abutment teeth. Already great strides have been made in this direction.

One of the first attachments for anterior bridges having this principle in mind was what has been variously called the Tinker crown, the three-quarter crown, the Carmichael crown, the staple crown or inlay, and I suppose other names. But regardless of name, most dentists are familiar with the principle of using the lingual, distal and mesial surfaces for the attachment of gold and I will not dwell on this.

I believe that most of us have been in the habit of using two of these open-faced crowns for a bridge, where one tooth is missing, so as to insure against the force of leverage on the dummy tooth. There seems to be no argument against this principle, but everything seems to go to prove that there should be insurance against this leverage that destroys bridges and teeth where only one tooth is used to support an adjoining dummy tooth.

It is with this in mind that I wish to offer an improvement on this type of bridge. At least I have never seen or read of this method being used.

We will assume that an upper lateral incisor is extracted. Make an open-face type of attachment on the adjoining cuspid. Now if there is not a cavity in the distal surface of the adjoining central incisor, cut a perfect approximal cavity for a gold foil filling. Extend well to lingual and gingival.

Now, having ideal access on account of the missing lateral incisor, insert a perfect foil filling and mallet well for condensation. While inserting the foil, contour the filling so that from the lingual surface there will be a cavity in the gold filling. Polish the filling and shape the cavity in it so that wax will draw from it from the lingual surface. See that there is a depression in the base of the cavity in the gold filling to give the resulting cast rest a firm seat.

Having shaped a cavity in the gold filling, make a wax pattern and cast an inlay to fit it, leaving the sprue extending from the inlay to solder to. This cast inlay rest may be cast of clasp gold to give strength. Place the attachment on the cuspid and the inlay rest in the cavity in the gold filling. The lug will be held securely in place if varnish or collodion is wiped in the cavity first.

Now, having the attachments in place, take the bite and an impression. The impression should be taken in com-plaster or some like material so that the impression will be easy to separate from model. Solder in a facing and polish, and you have a bridge that is cemented securely at one end and has a definite rest in a filling that has anchorage in undercuts and the griping of elastic dentin. The depression in the base of the cavity in the gold filling prevents the teeth from changing position.

The advantages of such a bridge are:

A translucent central incisor instead of an opaque one.

Less destruction of the central incisor than by other methods of fixed bridgework.

Slight mobility instead of rigidity.

A lighter and less bulky bridge; also the contour of the central incisor is the same as nature provided.

Being smaller, is cleaner.

The central having the gold filling and rest in the distal surface has a normal mesial surface and contact point, which is better than those we make, especially when appearance and susceptibility to decay of the approximal tooth is considered.

Less danger of resulting death of the pulp as the cavity in the central incisor is never exposed to saliva and thermal irritation.

In case of the bridge becoming uncemented, one end being a rest, the bridge is easily cemented in place again. —*Cosmos,* March, 1920.

